# Supplementary material for: Biogeography rather than substrate type determines bacterial colonization dynamics of marine plastics
Source: PeerJ. 2021 Sep 13;9:e12135. doi: 10.7717/peerj.12135 (PMC8445087; doi:10.7717/peerj.12135)
Supplement: Supplemental Information 1 — The environmental parameters were collected on 28th June (Cape Verde), 15th June (South Africa), 8th June (Japan) and 29th May (Chile) 2019. [file peerj-09-12135-s001.docx]

**Table X: Environmental parameters and site characteristics of each sampling site.** The environmental parameters were collected on 28^th^ June (Cape Verde), 15^th^ June (South Africa), 8^th^ June (Japan) and 29^th^ May (Chile) 2019.

| Chile | Japan | South Africa | Cape Verde | **Location** |
| --- | --- | --- | --- | --- |
| Punta de Choros, Coquimbo | Aikappu cape, Akkeshi Hokkaido | Muizenberg, Cape Town | Baia das Gatas, São Vincente | **Collection site** |
| 29°57'59.5"S 71°21'11.6"W | 43°01'14.3"N 144°50'12.0"E | 34°54'35.9"S, 18°25'10.2" E | 16°53'11.2"N 24°59'27.6"W | **Coordinates** |
| 30-33 | 30,3-31,9 | 35-37 | 36-40 | **Salinity range [psu]** |
| 14 | 12 | 14-17 | 27 | **Water temperature [°C]** |
| The coast from Coquimbo is influenced by the Humboldt current, a productive upwelling zone along the Chilean coast, transporting cold and nutrient rich waters. | The Akkeshi bay is influenced by the Oyashio current, transporting cold and nutrient rich waters from the north pole. | The coastal region of Cape Town is part of the Benguela Upwelling system, characterized by cold and nutrient rich waters being transported to the surface throughout the year with only little fluctuation. | The island of São Vincente is located between cold subtropic and tropical warm water masses. Furthermore, the coastal waters can be influenced by terrestrial dust particles from Europe and the Sahara Desert. | **Site characteristics** |
