# Supplementary material for: Biogeography rather than substrate type determines bacterial colonization dynamics of marine plastics
Source: PeerJ. 2021 Sep 13;9:e12135. doi: 10.7717/peerj.12135 (PMC8445087; doi:10.7717/peerj.12135)
Supplement: Supplemental Information 4 — The blastn suite (blastn. Bethesda (MD): National Library of Medicine (US), National Center for Biotechnology Information; 2004–2021. Available from https://blast.ncbi.nlm.nih.gov/Blast.cgi?PROGRAM=blastn&PAGE_TYPE=BlastSearch&BLAST_SPEC=&LINK_LOC=blasttab&LAST_PAGE=blastp) was employed with the rRNA/ITS database (Sayers et al. 2020). [file peerj-09-12135-s004.docx]

**Supplementary table SX:** **Taxonomic identification of the 18 ASVs shared across three locations by the NCBI blast algorithm**. The blastn suite (blastn. Bethesda (MD): National Library of Medicine (US), National Center for Biotechnology Information; 2004-2021. Available from <https://blast.ncbi.nlm.nih.gov/Blast.cgi?PROGRAM=blastn&PAGE_TYPE=BlastSearch&BLAST_SPEC=&LINK_LOC=blasttab&LAST_PAGE=blastp>)was employed with the rRNA/ITS database (Sayers *et al.* 2020).

| **ASV hash** | **Taxonomic identification (best hit)** | **Percent identity [%]** | **Query cover [%]** |
| --- | --- | --- | --- |
| 3439d5c6b96c640c6517f25ef7d425d1 | *Kordia ulvae* strain SC2 | 100 | 100 |
| b4947c2533dc74faaff4a7fee409d4cd | *Woeseia oceani* strain XK5 | 99.64 | 99 |
| 3175bd423293fd694db07c69f873c6c6 | *Altererythrobacter aquaemixtae* strain JSSK-8 | 98.52 | 100 |
| 24626eb258299d13b81d7bc48b30c7ad | *Kordiimonas aquimaris* strain MEBiC06554 | 97.78 | 100 |
| ae7b53df4ed45f7aa2e8cde29d8f1ae1 | *Portibacter lacus* strain YM8-076 | 100 | 100 |
| a2e2301eb2c4bb9f5f5df5be987bf39e | *Lutibacter profundi* strain LP1 | 97.04 | 100 |
| e47f9c6dd1439efd4276a8c3f78cb6b0 | *Pseudoalteromonas denitrificans* strain Nygaard 1977 | 99.26 | 100 |
| 7df4f98dba18ab55a38016acdf78aa9a | *Gramella aquimixticola* strain HJM-19 | 92.22 | 100 |
| 1c85a75dbc4fb384c65c4de5fd5ad3c6 | *Phycisphaera mikurensis* NBRC 102666 | 85.38 | 93 |
| 03d722eddf542c1523e4e362be158784 | *Portibacter lacus* strain YM8-076 | 93.49 | 96 |
| 83fd37042e7c2fc0fc21d33722752a5a | *Reichenbachiella agariperforans* strain KMM 3525 | 97.41 | 100 |
| 3e601031df694c477a11d85ba1c88cc5 | *Fabibacter misakiensis* | 94.81 | 100 |
| 4d4109444303bf03ab42c209b1af9654 | *Woeseia oceani* strain XK5 | 96.25 | 98 |
| 9e2dddf161dc96fe080039e214d14eae | *Ilumatobacter nonamiensis* YM16-303 | 99.63 | 100 |
| f6e5d25300c1296fbcae555696f123bd | *Nitrospina gracilis* 3/211 | 85.61 | 97 |
| 51325216cbe31a19d795004175d99db9 | *Haloferula chungangensis* strain CAU 1074 | 98.15 | 100 |
| eb57abc1875a88b873a0e4a337bcea56 | *Lysobacter spongiae* strain 119BY6-57 | 90.41 | 100 |
| 710dd11aa66320124b32ab181c34d4e0 | *Kiritimatiella glycovorans* strain L21-Fru-AB | 84.44 | 98 |

Sayers, E. W., Beck, J., Bolton, E. E., Bpurexis, D., Brister, J. R., Canese, K., Comeau, D. C., Funk, K., Kim, S., Klimke, W., Marchler-Bauer, A., Landrum, M., Lathrop, S., Lu, Z., Madden, T. L., O’Leary, N, Phan, L., Rangwala, S. H., Schneider, V. A., Skripchenko, Y., Wang, J., Ye, J., Trawick, B. W., Pruitt, K. D., & Sherry, S. T. (2021). Database resources of the National Center for Biotechnology Information. *Nucleic Acids Research*, 8(49), D10-D17. Doi: 10.1093/nar/gkaa892
