# Supplementary figures and images for: Biogeography rather than substrate type determines bacterial colonization dynamics of marine plastics
Source: PeerJ. 2021 Sep 13;9:e12135. doi: 10.7717/peerj.12135 (PMC8445087; doi:10.7717/peerj.12135)

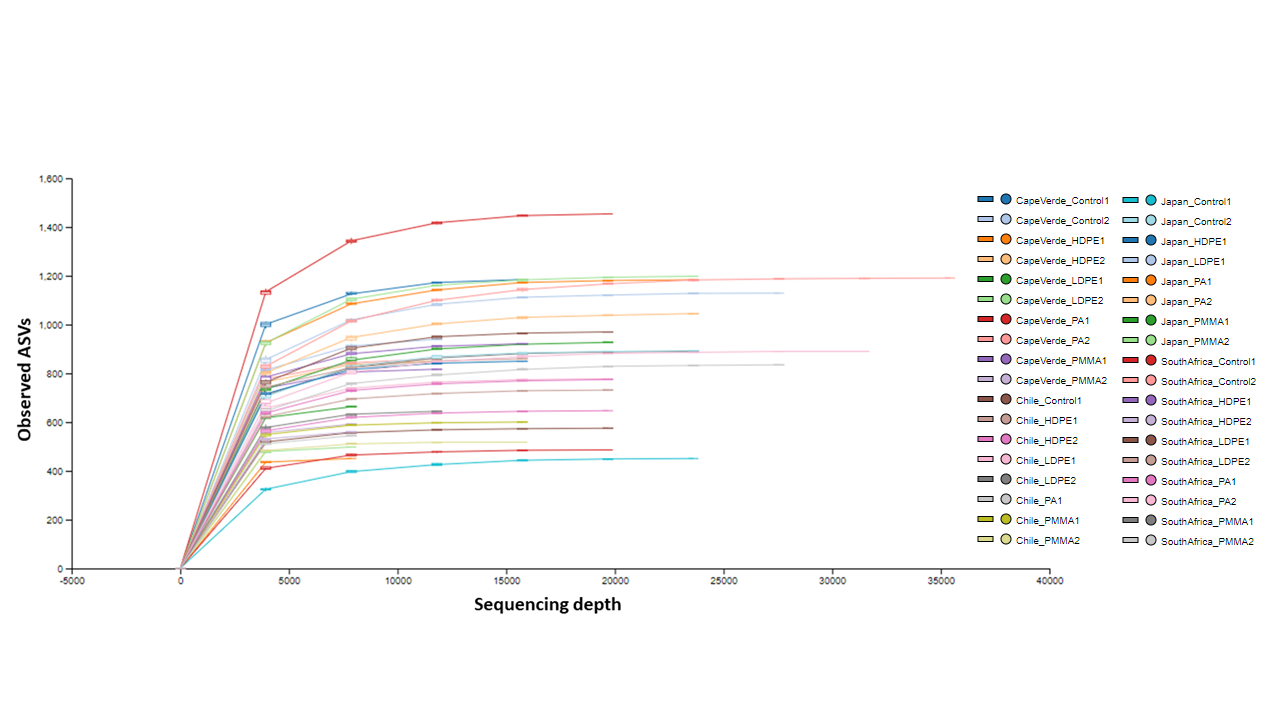

Supplement: Supplemental Information 5 — Alpha rarefaction curves displaying the absolute number of ASVs present in each sample for all coastal samples. [file peerj-09-12135-s005.png]

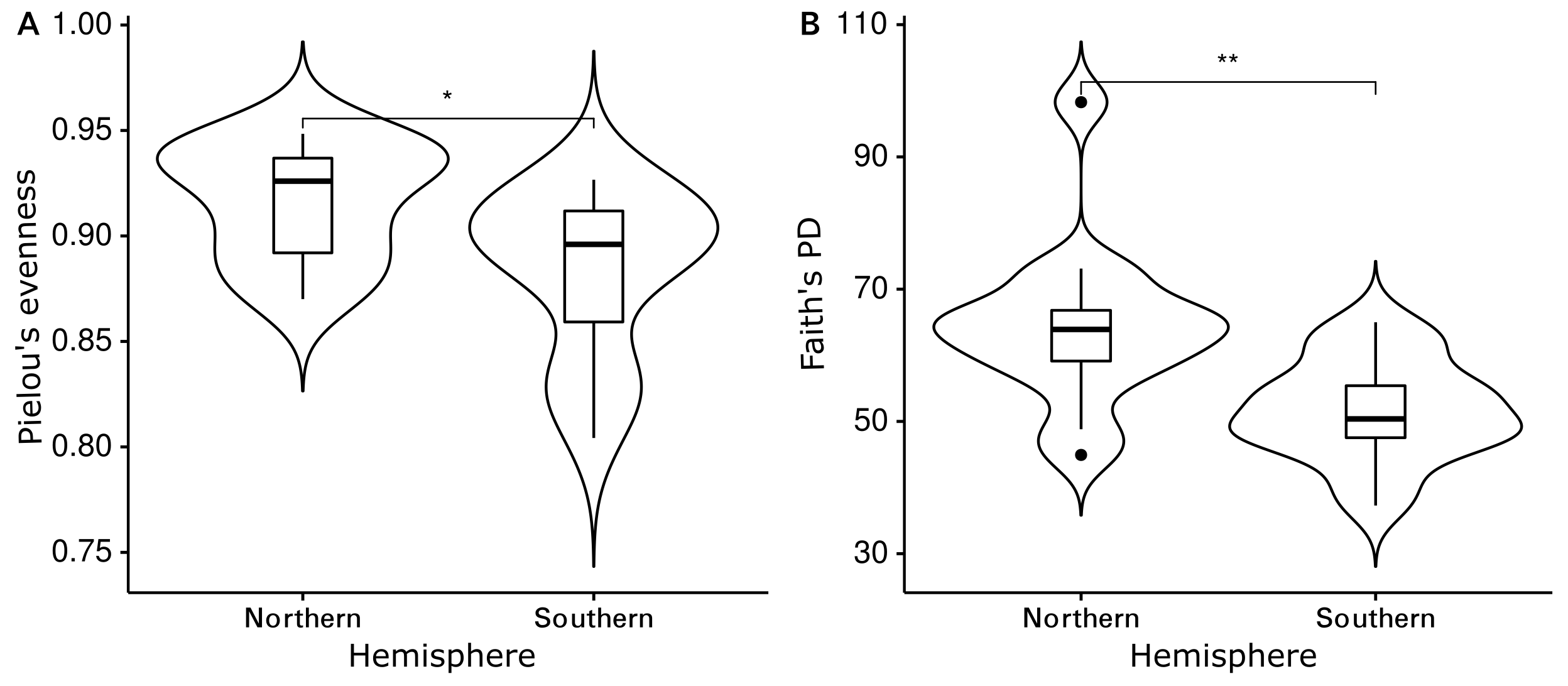

Supplement: Supplemental Information 6 — Violin plots depicting (A) Pielou’s evenness (p = 0.018) and (B) Faith’s PD (p = 0.001) of plastic replicates within each hemisphere (N = 29): Northern Hemisphere (n = 14), Southern Hemisphere (n = 15). Significant results are depicted with symbolic number coding, where * and ** represent Wilcoxon p-values ≤ 0.05 and ≤ 0.01, respectively. [file peerj-09-12135-s006.png]

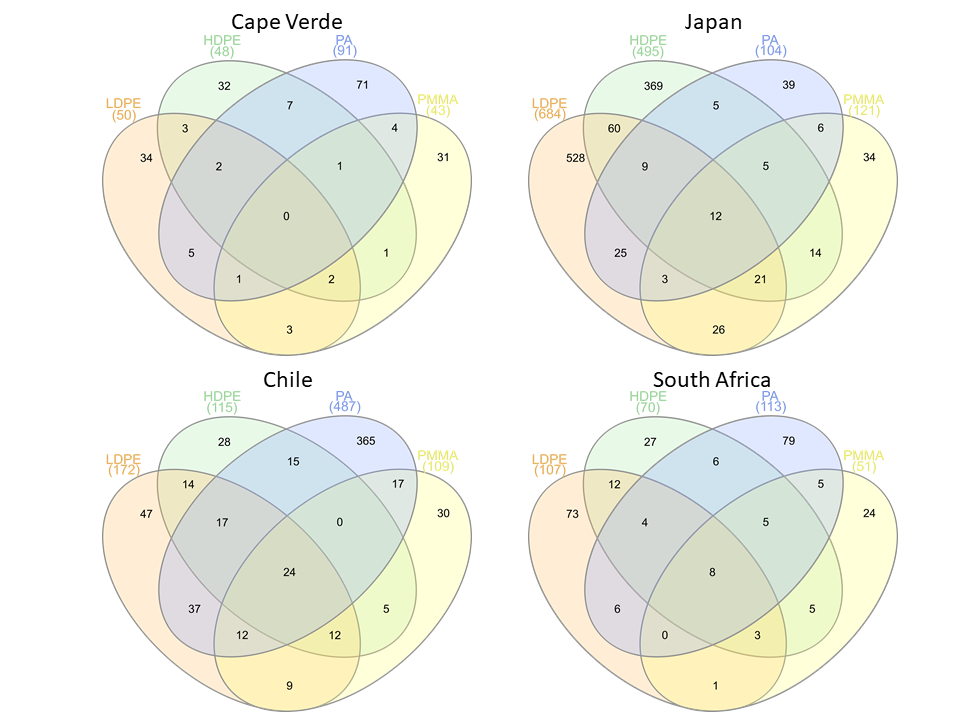

Supplement: Supplemental Information 7 — All ASVs unique per polymer type were used, regardless of read count, while ASVs also found in the glass controls were subtracted. The Venn diagrams were drawn with InteractiVenn (Heberle et al., 2015). [file peerj-09-12135-s007.png]
