# Supplementary material for: Biogeography rather than substrate type determines bacterial colonization dynamics of marine plastics
Source: PeerJ. 2021 Sep 13;9:e12135. doi: 10.7717/peerj.12135 (PMC8445087; doi:10.7717/peerj.12135)
Supplement: Supplemental Information 8 [file peerj-09-12135-s008.docx]

**Supplementary file SX. Qiime 2 pipeline as used in this study.**

# Remove heterogeneiety spacers & primers

# $PWD = “Print working directory”

source activate qiime2-2018.11

cd $PWD/

mkdir trimmed

cd $PWD/reads/

for i in *_R1_001.fastq

do

cutadapt -g CCTACGGGAGGCAGCAG -o $PWD/trimmed/$i $i

done

# Sequence quality check

cd $PWD/

mkdir dada2

qiime tools import \

--type 'SampleData[SequencesWithQuality]' \

--input-path $PWD/pre-33-manifestV34.csv \

--output-path $PWD/dada2/single-end-demux.qza \

--input-format SingleEndFastqManifestPhred33

qiime demux summarize \

--i-data $PWD/dada2/single-end-demux.qza \

--o-visualization $PWD/dada2/single-end-demux.qzv

# Trimming, truncation, chimera removal & DADA2 algorithm

qiime dada2 denoise-single \

--i-demultiplexed-seqs$PWD/dada2/single-end-demux.qza \

--p-trim-left 0 \

--p-trunc-len 270 \

--p-chimera-method consensus \

--p-n-threads 10 \

--p-n-reads-learn 1000000 \

--o-table $PWD/dada2/dada2-table.qza \

--o-representative-sequences $PWD/dada2/dada2-rep-seqs.qza \

--o-denoising-stats $PWD/dada2/dada2-denoising-stats.qza

# Visualize denoising stats & preliminary feature table

qiime metadata tabulate \

--m-input-file $PWD/dada2/dada2-denoising-stats.qza \

--o-visualization $PWD/dada2/dada2-denoising-stats.qzv

qiime feature-table summarize \

--i-table $PWD/dada2/dada2-table.qza \

--o-visualization $PWD/dada2/dada2-table.qzv \

--m-sample-metadata-file $PWD/metadataV34.txt

# Taxonomic classification

# $PWD for –i-classifier should lead to the classifier database

qiime feature-classifier classify-sklearn \

--p-n-jobs 1 \

--p-reads-per-batch 1000 \

--i-classifier $PWD/Silva132V3V4-classifier.qza \

--i-reads $PWD/dada2/dada2-rep-seqs.qza \

--p-confidence 0.8 \

--o-classification $PWD/dada2/Silva132V3V4-taxonomy-dada2.qza

# Check how many sequences are unassigned

qiime metadata tabulate \

--m-input-file $PWD/dada2/Silva132V3V4-taxonomy-dada2.qza \

--o-visualization $PWD/dada2/Silva132V3V4-taxonomy-dada2.qzv

# Remove Chloroplasts, Mitochondria, Unassigned, etc.

qiime feature-table filter-features \

--i-table $PWD/dada2/dada2-table.qza \

--m-metadata-file $PWD/dada2/Silva132V3V4-taxonomy-dada2.qza \

--p-where "Taxon NOT LIKE '%Chloroplast%'" \

--o-filtered-table $PWD/dada2/dada2-tablenochloroplasts.qza

qiime feature-table filter-features \

--i-table $PWD/dada2/dada2-tablenochloroplasts.qza \

--m-metadata-file $PWD/dada2/Silva132V3V4-taxonomy-dada2.qza \

--p-where "Taxon NOT LIKE '%Mitochondria%'" \

--o-filtered-table $PWD/dada2/dada2-tablenochloroplastsmitochondria.qza

qiime feature-table filter-features \

--i-table $PWD/dada2/dada2-tablenochloroplastsmitochondria.qza \

--m-metadata-file $PWD/dada2/Silva132V3V4-taxonomy-dada2.qza \

--p-where "Taxon NOT LIKE '%Unassigned%'" \

--o-filtered-table$PWD/dada2/dada2-tablenochloroplastsmitochondriaUnassigned.qza

qiime feature-table summarize \

--i-table $PWD/dada2/dada2-tablenochloroplastsmitochondriaUnassigned.qza \

--o-visualization $PWD/dada2/dada2-tablenochloroplastsmitochondriaUnassigned.qzv \

--m-sample-metadata-file $PWD/metadataV34.txt

# Subsample based on sequence frequency

qiime feature-table filter-samples \

--i-table $PWD/dada2/dada2-tablenochloroplastsmitochondriaUnassigned.qza \

--p-min-frequency 5700 \

--o-filtered-table $PWD/dada2/dada2-tablenochloroplastsmitochondriaUnassigned5700.qza

qiime feature-table summarize \

--i-table $PWD/dada2/dada2-tablenochloroplastsmitochondriaUnassigned5700.qza \

--o-visualization $PWD/dada2/dada2-tablenochloroplastsmitochondriaUnassigned5700.qzv \

--m-sample-metadata-file $PWD/metadataV34.txt

# Barplots

qiime taxa barplot \

--i-table $PWD/dada2/dada2-tablenochloroplastsmitochondriaUnassigned5700.qza \

--i-taxonomy $PWD/dada2/Silva132V3V4-taxonomy-dada2.qza \

--m-metadata-file $PWD/metadataV34.txt \

--o-visualization $PWD/dada2/taxa-bar-plots.qzv

# Create phylogenetic backbone tree

qiime phylogeny align-to-tree-mafft-fasttree \

--p-n-threads 10 \

--i-sequences $PWD/dada2/dada2-rep-seqs.qza \

--o-alignment $PWD/dada2/aligned-rep-seqs.qza \

--o-masked-alignment $PWD/dada2/masked-aligned-rep-seqs.qza \

--o-tree $PWD/dada2/unrooted-tree.qza \

--o-rooted-tree $PWD/dada2/rooted-tree.qza

# Downstream analysis

qiime diversity core-metrics-phylogenetic \

--p-n-jobs 8 \

--i-phylogeny $PWD/dada2/rooted-tree.qza \

--i-table $PWD/dada2/dada2-tablenochloroplastsmitochondriaUnassigned5700.qza \

--p-sampling-depth 5700 \

--m-metadata-file $PWD/metadataV34.txt \

--output-dir $PWD/dada2/core-metrics-results

qiime diversity alpha-rarefaction \

--i-table $PWD/dada2/dada2-tablenochloroplastsmitochondriaUnassigned5700.qza \

--i-phylogeny $PWD/dada2/rooted-tree.qza \

--p-max-depth 5700 \

--m-metadata-file $PWD/metadataV34.txt \

--o-visualization $PWD/dada2/alpha-rarefaction.qzv
